# Proprioception in the cerebellum

**DOI:** 10.3389/fnhum.2014.00212

**Published:** 2014-04-09

**Authors:** Matthieu P. Boisgontier, Stephan P. Swinnen

**Affiliations:** Movement Control and Neuroplasticity Research Group, Biomedical Sciences Group, Department of KinesiologyKU Leuven, Leuven, Belgium

**Keywords:** cerebellum, internal models, kinesthesia, perception, proprioception, statesthesia

Proprioception is the ability to interpret our musculo-skeletal state (e.g., position and movement) by processing information originating from our own body. While it is generally accepted that passive proprioception (i.e., proprioception in the absence of muscle contraction) is dependent only on the processing of peripheral inputs, the precise nature of the processes constituting active proprioception (i.e., proprioception with muscle contraction) is still not clear (Proske and Gandevia, 2012). Central to this knowledge gap is the difficulty in accurately determining the processes responsible for the improvement of proprioception in active compared to passive movements (e.g., Fuentes and Bastian, 2010). This improvement is assumed to potentially result from [1] enhanced peripheral muscle information (through gamma motoneurons of the muscle spindles), [2] the direct transmission of a copy of motor commands (i.e., efference copy) from motor to sensory processing areas, and/or [3] the involvement of predictive models through the cerebellum (Wolpert and Miall, 1996; Bhanpuri et al., 2012; Proske and Gandevia, 2012). Here, based on the results of Bhanpuri et al. (2013), we propose that cerebellar predictive models can *fully* account for the improvement of proprioception in active movements. We also describe the cellular arrangement that may underlie the involvement of predictive models in proprioception.

In their recent study, Bhanpuri et al. (2013) tested proprioception in cerebellar patients and well matched controls. They measured perceptual thresholds of the dominant arm using an exoskeleton robot system in three proprioceptive tasks: passive, active (active-simple), and active with a complex pattern of resistive torque during forearm displacement making this displacement unpredictable (active-complex). Based on Weber fractions, controls demonstrated better proprioception in the active-simple task compared to the passive (*p* < 0.005) and active-complex tasks (*p* < 0.022), which were not different from each other (*p* > 0.38). In contrast, no differences were found between any of the tasks in cerebellar patients (all *p* > 0.28), who performed worse than controls in the active-simple task (*p* < 0.03).

The similar performance observed in the passive and active-complex task in controls reveals that the proprioceptive benefit usually observed in active compared to passive conditions is neither related to an enhancement of information from the peripheral muscle [1] nor to the direct transmission of an efference copy from motor to sensory areas [2]. Indeed, these two processes may have been operating in the active-complex task but did not improve proprioceptive performance compared to the passive task. Furthermore, the absence of improvement in the active-simple task compared to the passive task in cerebellar patients demonstrates that the improved proprioception observed in the active-simple task in controls can be fully accounted for by a process located in the cerebro-cerebellar loop. Finally, cerebellar patients demonstrated a lower performance level than controls in the active-simple (predictable) task but not in the active-complex (non-predictable) task. Therefore the process that is most likely responsible for the improvement in proprioception is predictive modeling, which is thought to be supported by the cerebellum [3].

A recent study in mice demonstrates that individual granule cells in the cerebellum can mix proprioceptive afferents from the spinocerebellar tract and efference copy from the cerebral cortex (Huang et al., 2013). This multimodal arrangement provides the anatomical basis for converting motor corollary discharges into sensory coordinates. Indeed, granule cells can generate action potentials in response to a single input (Rancz et al., 2007). Therefore, sensory and motor-related inputs can potentially substitute for one another to fire a granule cell. The nature of the granule cells' output is still unknown. However, studies testing experimental phantom limbs by blocking peripheral nerves revealed conscious sensations of limb movement in the absence of any sensory input when subjects attempted to move (e.g., Gandevia et al., 2006). This result clearly supports a conversion of the efference copy from motor to sensory coordinates making proprioceptive prediction possible.

So far, the literature has emphasized the role of predictive models in the control of movement where intended actions are compared with actual actions to generate error signals. However, results from Bhanpuri et al. (2013) as well as studies of phantom limbs demonstrate that the purpose of cerebellar processes is not only motor control but also proprioception *per se*.

## Conflict of interest statement

The authors declare that the research was conducted in the absence of any commercial or financial relationships that could be construed as a potential conflict of interest.
